# Supplementary material for: A Novel Homozygous Nonsense Variant in the DYM Underlies Dyggve-Melchior-Clausen Syndrome in Large Consanguineous Family
Source: Genes (Basel). 2023 Feb 17;14(2):510. doi: 10.3390/genes14020510 (PMC9956627; doi:10.3390/genes14020510)
Supplement: Supplementary file 1 [file genes-14-00510-s001.zip › 7. Table S1 supplementary.pdf]

Table S1: Primers sequences used to amplify and sequence DYM coding regions

Primer Name	Forward (5'-3')	Reverse (5'-3')
Exon_2_DYM	CTGGCCTAGCCCCATATT	TCTTTTGGATTTTCTACTGATCTATG
Exon_3_DYM	CCCCAAGCTCTTCTCTTTAGG	ATGGCTTAGCAGAGTCCATTACC
Exon_4_DYM	GCAGCAGTTTCTCTCATTGC	TCTCACCCCAGTGACTTAGAC
Exon_5_DYM	AGGATGGACAAAACGGTTG	CTTCAACCCCACCAGCTT
Exon_6_DYM	CGTACAGGAGCCTAAAGTACC	CAGAGATTAATTCTGTGCCTACC
Exon_7_DYM	GATAACTAACCCAAATACACCAGC	CTATGCACTGGGTTTAAAGAGAC
Exon_8_DYM	TGCAGTGCTCAATTTCTGTGAG	CAGCTGCACAAATTCAATGTAAC
Exon_9_DYM	CTGGCCTTGTGAAATGTATAGAC	GGATCAGATATAAAGGATGCTTG
Exon_10_DYM	GAATCTGAAATGTAGTGACTTTCC	ACGAGTTCCTGGGTGCAGC
Exon_11_DYM	GAGCCTAAATGGTCCTTGCAG	GGTTCTCACAGGAACATCTACCA
Exon_12_DYM	ATTTGCGTGGCCTCTGTCT	TGTCACGAGAATTTTAGCATCC
Exon_13_DYM	GGGGCTATTGCAGATACACATT	AAGATGGATACTATGGTGGTTCCT
Exon_14_DYM	CTTGCTGTTAACACAGGCATGAA	ACAGTAAAATCTTGAGAAGAGAGGG
Exon_15_DYM	TGAGCTTCTTCTCTTCATGATCT	ACACACACAGATTACCACAACTAT
Exon_16_DYM	GTGAATTAAAAGCCGGGTCA	TGGCACTGAGTCACACTATGG
Exon_17_DYM	ACCAGGACCCATTGTGTTACT	TAAAGAGGATTGCACGAGTGTGT
