# Supplementary figures and images for: A Novel Homozygous Nonsense Variant in the DYM Underlies Dyggve-Melchior-Clausen Syndrome in Large Consanguineous Family
Source: Genes (Basel). 2023 Feb 17;14(2):510. doi: 10.3390/genes14020510 (PMC9956627; doi:10.3390/genes14020510)

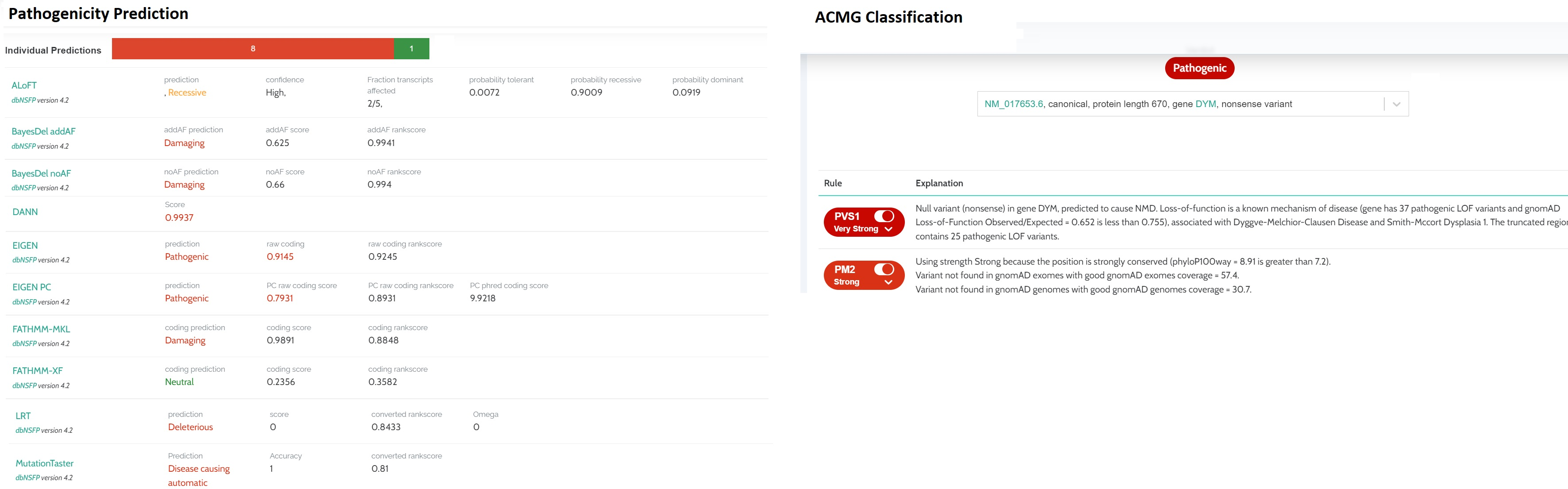

Supplement: Supplementary file 1 [file genes-14-00510-s001.zip › 6. Figure S1 supplementary.tif]
